# Emergency department nurse-led systematic nursing model for septic shock: impact on 1-hour bundle adherence and mortality

**DOI:** 10.3389/fmed.2026.1967565

**Published:** 2026-10-27

**Authors:** Suxiang Wei, Xueyan Li, Xiusong Wang, Tingting Ke, Caili Lin, Yue Zhao, Huihui Sun

**Affiliations:** Emergency Department, The First Affiliated Hospital of Wenzhou Medical University, Wenzhou, China

**Keywords:** 1-hour bundle, adherence, Delphi method, emergency department, mortality, septic shock, systematic nursing model

## Abstract

**Objective:**

To evaluate the impact of a systematic septic shock nursing model led by emergency department (ED) nurses on adherence with resuscitation protocols and clinical outcomes in patients with septic shock.

**Methods:**

Our team employed the Delphi method to develop a systematic nursing model, and it was implemented in August 2023. To verify its application effect, a retrospective controlled study was conducted in the emergency resuscitation room of the First Affiliated Hospital of Wenzhou Medical University. Patients who met the Sepsis-3 criteria for septic shock were consecutively enrolled from January 2022 to February 2025. According to the nursing model received during different periods, they were divided into two groups: the control group (enrolled from January 2022 to July 2023, *n* = 331) received conventional nursing, and the observation group (enrolled from August 2023 to February 2025, *n* = 343) received the systematic nursing model led by emergency nurses. Propensity score matching (1:1, caliper value 0.2) was used to balance differences. Primary outcomes included the 1-hour bundle adherence rate, 28-day and 90-day mortality, and 24-hour resuscitation success rate. Secondary outcomes included SOFA score, duration of vasoactive agent use, mechanical ventilation duration, ICU length of stay, and incidence of adverse events.

**Results:**

After propensity score matching, 524 patients were successfully matched, with 262 patients in each group. The 1-hour bundle adherence rate was significantly higher in the observation group than in the control group (23.66% vs. 14.12%, *p* = 0.005), although the absolute adherence rate remained low in both groups. Compared with the control group, the observation group showed a significant decreases in 28-day and 90-day mortality, and a significant increase in the 24-hour resuscitation success rate (*p* < 0.05). Moreover, SOFA scores were significantly reduced, and both the duration of vasoactive agent use and ICU length of stay were shortened (*p* < 0.05). The incidence of adverse events was also significantly lower in the observation group (*p* < 0.05).

**Conclusion:**

The systematic nursing model led by ED nurses significantly improves adherence in patients with septic shock, reduces short and medium-term mortality, enhances organ function recovery, and shortens ICU length of stay, demonstrating good potential for clinical implementation.

## 1 Introduction

Sepsis and septic shock remain leading causes of death and disability worldwide. There were approximately 48.9 million incident cases of sepsis globally in 2020, with 11 million deaths (1, 2). Despite substantial progress in understanding pathophysiology over the past two decades, the mortality rate of septic shock remains high, with in-hospital mortality exceeding 40% (3, 4). The 1-hour sepsis bundle, including lactate measurement, blood cultures before antibiotics, broad-spectrum antibiotics, crystalloid resuscitation, and vasopressors when indicated, is a core quality indicator recommended by the Surviving Sepsis Campaign (3). However, adherence to this bundle remains low in many clinical settings, and delayed or missed adherence is consistently associated with adverse outcomes (5, 6).

Multidimensional quality improvement interventions, such as electronic alerts, rapid response teams, standardized checklists, and audit-feedback programs, have been shown to improve sepsis care and clinical outcomes (7–9). Nevertheless, most existing studies have focused on ICU settings or single intervention components. Evidence on systematically integrated, emergency department (ED) nurse-led nursing models for septic shock remains limited (10).

To address this gap, our institution developed a systematic ED nurse-led septic shock nursing model using the Delphi method. This model integrates nurse-triggered electronic alerts, a sepsis rapid response team, a standardized 1-hour bundle checklist, information-platform-based collaboration, and dynamic monitoring. While previous studies have evaluated individual components or ICU-based programs, this retrospective propensity-matched study specifically assessed the impact of this integrated, ED nurse-led model on 1-hour bundle adherence and short- and medium-term mortality in patients with septic shock in the emergency resuscitation room.

## 2 Research subjects and methods

### 2.1 Research subjects

A retrospective study design was adopted. Patients who met the Sepsis-3 criteria for septic shock (11, 12) were consecutively enrolled in the emergency resuscitation room of the First Affiliated Hospital of Wenzhou Medical University from January 2022 to February 2025. According to the different nursing models, it was divided into conventional nursing (the control group, *n* = 331) and the systematic nursing model led by emergency nurses (the observation group, *n* = 343. After propensity score matching (PSM), 524 patients were successfully matched, with 262 patients in each group for subsequent analysis (see Figure 1). The study was approved by the Ethics Committee of the First Affiliated Hospital of Wenzhou Medical University (approval number: KY2026-R016), and the requirement for informed consent was waived.

**Figure 1:**
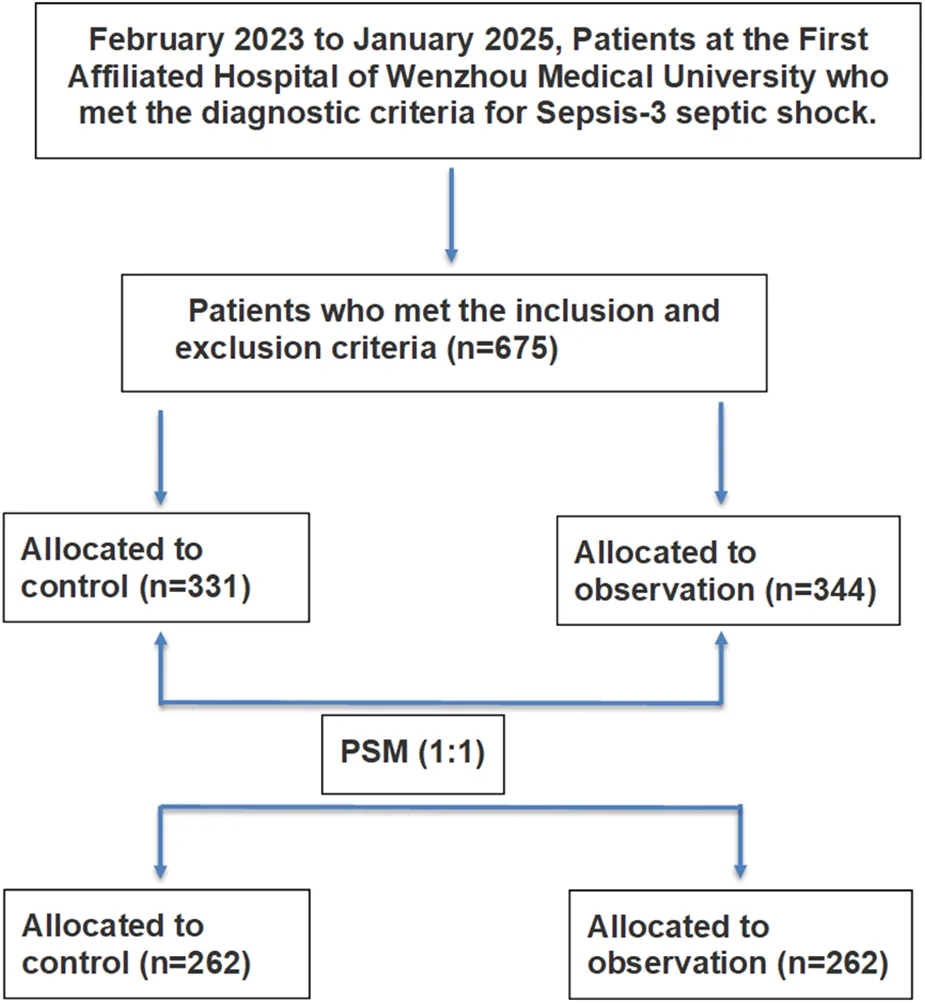
PSM process.

This study was conducted in the emergency resuscitation room of the First Affiliated Hospital of Wenzhou Medical University, a tertiary care, university-affiliated general hospital serving Wenzhou and the surrounding areas. The emergency department mainly serves adult urban and rural residents and commonly attends patients with trauma, cardiovascular and cerebrovascular emergencies, respiratory and gastrointestinal diseases, sepsis/septic shock, and poisoning.

Inclusion Criteria: 1) Meeting the Sepsis-3 consensus criteria for septic shock (11, 12): (a) confirmed or suspected infection; (b) requirement of vasopressors to maintain a mean arterial pressure (MAP) ≥ 65 mmHg after adequate fluid resuscitation; (c) serum lactate level >18 mg/dL; 2) Receiving initial treatment in the emergency resuscitation room.

Exclusion Criteria: 1) Pregnant women; 2) Patients with end-stage disease and an estimated survival <24 h; 3) Patients with a documented “do not resuscitate” order on admission; 4) Patients who had already received complete 1-hour bundle therapy prior to transfer from other healthcare institutions; 5) Patients whose primary cause of death was non-infectious, such as trauma, extensive burns, or cerebral hemorrhage.

### 2.2 Patient identification and enrollment process

A three-step approach combining automatic identification with manual review was used to screen patients with septic shock. 1) A combined electronic screening trigger integrated into the hospital information system was used at the triage entry point, based on qSOFA, lactate, and MAP. The alert was triggered by any of the following: qSOFA ≥2; lactate >18 mg/dL with MAP <65 mmHg; or infection-related order sets. NEWS was not used because its required SpO₂ and oxygen therapy data are not consistently available at the triage entry point. The alert was simultaneously received at the nursing station. 2) The emergency department nurse on duty performed a nursing assessment within 10 min and notified the physician to confirm the diagnosis. 3) Research team members verified the inclusion and exclusion criteria and completed enrollment registration.

### 2.3 Research methods

#### 2.3.1 Construction of the systematic septic shock nursing model led by ED nurses

The systematic nursing model was developed using the Delphi method (13). Core items of the care model were preliminarily formulated through literature review and research group discussion. Twenty experts from the fields of emergency critical care, nursing management, infection control, and related specialties were selected for two rounds of expert consultation. The experts should have at least 10 years of working experience, hold a mid-level or higher professional title, and show a strong interest in the research topic. Both survey rounds achieved 100% response, with corresponding Cr values of 0.87 and 0.89. Mean importance score >3.5, coefficient of variation (CV) < 0.25, and Kendall's W coefficient of concordance with statistical significance (*p* < 0.05). All constructed measures met the retention criteria, as shown in Table 1.

**Table 1:** The systematic septic shock care model led by emergency department nurses.

Sections	Nursing measures	Responsible person	Tools/Methods	Importance scores	CV
Quick Response Team and Alarm System	1. Establish an SRRT led by emergency nurses, with the head nurse serving as the coordinator. The members include senior emergency nurses, emergency doctors, ICU consultation doctors, laboratory liaison officers, and pharmacy liaison officers.	Nursing team leader	SRRT organizational structure and responsibility assignment	4.61 ± 0.55	0.148
	2. The nursing staff undergo a 2-week systematic training (including Sepsis-3, SSC 2021 guidelines, 1-hour bundle care, alarm response, and multidisciplinary communication), and must pass both a written test (≥85 points) and an operational assessment.	Nursing manager/trainer	Standardized training curriculum and dual-assessment system	4.39 ± 0.60	0.125
	3. SRRT operates on a 24/7 basis. Once the nurse receives the call, they will conduct a preliminary assessment within 5 min and notify the team. The core members will be on-site at the bedside within 15 min.	Bedside nurse/all SRRT members	Response time recording form	4.78 ± 0.50	0.208
	4. A dedicated alarm terminal is set up at the nursing station, which automatically calculates qSOFA/SOFA by interfacing with HIS/LIS; the triggering conditions are (qSOFA≥2, lactate>2 and MAP<65, infection treatment order); the nursing records must register the three time points of “alarm reception-assessment completion-doctor notification”.	Bedside nurse	Electronic alert system; time-point recording form	4.20 ± 0.38	0.118
Information Collaboration Platform	1. The nursing team leads the development of an integrated information platform covering admission office, radiology, laboratory, pharmacy, and physicians.	Nursing team/information technology department	Integrated information collaboration platform	4.52 ± 0.58	0.131
	2. Nurses use mobile handheld terminals (PDA) to scan patient wristbands to record vital signs, confirm medication administration, and verify specimen collection; the entire process is traceable.	Bedside nurse	PDA, wristband scanning system	4.46 ± 0.44	0.123
Standardized Bundle Therapy and Dynamic Monitoring	1. Use the 1-hour bundle nursing checklist; the bedside nurse executes each item, records completion time, and the physician confirms with dual signature.	Bedside nurse+physician	1-hour bundle nursing checklist (dual signature)	4.56 ± 0.56	0.115
	2. Lactate measurement: blood sampling and submission within 15 min.	Bedside nurse	Blood specimen collection protocol	4.40 ± 0.48	0.122
	3. Blood culture collection before antibiotics: bilateral double-bottle collection within 30 min.	Bedside nurse	Blood culture collection protocol	4.47 ± 0.46	0.108
	4. Broad-spectrum antibiotic administration: prepare and infuse after verifying the order, ensuring completion within 1 h.	Bedside nurse	Antibiotic infusion workflow	4.65 ± 0.50	0.119
	5. Crystalloid resuscitation: for patients with hypotension or lactate ≥36 mg/dL, establish dual-channel intravenous infusion, calculate total volume at 30 mL/kg, and control infusion rate.	Bedside nurse (under physician's order)	Dual-channel infusion pump, fluid calculation sheet	4.58 ± 0.52	0.11
	6. Vasoactive agent administration: prepare norepinephrine, establish central/peripheral venous access, continuously monitor MAP, and adjust infusion rate to maintain MAP ≥65 mmHg.	Bedside nurse (under physician's order)	Norepinephrine infusion protocol, MAP monitor	4.73 ± 0.52	0.114
	7. Bedside ultrasound to assess inferior vena cava collapsibility index (nurse-assisted under physician guidance after training), and monitor lactate clearance every 2 h, with timely feedback to the physician.	Nurse (under physician guidance)	Bedside ultrasound, lactate monitoring record sheet	4.59 ± 0.54	0.126
	8. Precise fluid input/output recording: use electronic urine output monitor and micro-infusion pump system, with hourly summary of fluid balance.	Bedside nurse	Electronic urine output monitor, micro-infusion pump, fluid balance sheet	4.54 ± 0.47	0.133
Infection Prevention and Zonal Management	1. On arrival at triage, the nurse checks the patient's health code status using the Zhejiang Health Code system.	Triage nurse	Health Code system	4.37 ± 0.49	0.12
	2. For patients with abnormal health codes, immediately activate the infection prevention protocol: transfer to an independent isolation resuscitation unit, prepare dedicated resuscitation cart and protective supplies, assign a dedicated nurse for isolation care, and document the zonal management in nursing records.	Triage/dedicated nurse	Independent isolation resuscitation unit, infection prevention protocol, isolation nursing record sheet	4.61 ± 0.44	0.117
Continuous Nursing Quality Improvement	1. Establish a hospital-level sepsis nursing quality improvement working group (headed by the deputy director of nursing, co-headed by the head nurse of emergency department; members include emergency nursing team leaders, quality management nursing representatives, and infection control nurses).	Deputy director of nursing/head nurse of emergency department	Working group organizational structure	4.41 ± 0.51	0.113
	2. Hold routine meetings every two weeks and quality analysis meetings monthly.	All working group members	Meeting system and records	4.57 ± 0.49	0.128
	3. Apply the PDCA cycle to conduct root-cause analysis on delayed adherence to the 1-hour bundle nursing protocol, propose improvement plans, and verify effectiveness in the next cycle.	All working group members	PDCA management tool, root-cause analysis form	4.85 ± 0.58	0.122
	4. The working group is also responsible for continuing education and re-certification of SRRT nursing members.	Working group members	Training and re-certification assessment system	4.52 ± 0.54	0.135

ED, emergency department; SRRT, sepsis rapid response team; ICU, intensive care unit; HIS, hospital information system; LIS, laboratory information system; qSOFA, quick Sequential Organ Failure Assessment; SOFA, Sequential Organ Failure Assessment; MAP, mean arterial pressure; PDA, personal digital assistant; PDCA, Plan-Do-Check-Act; CV, coefficient of variation; SSC, Surviving Sepsis Campaign; SD, standard deviation. Importance scores are presented as mean ± SD.

After the first round of consultation, 2 items were removed, 1 group of similar items was merged, and 1 new item was added. After the second round of consultation, all items met the retention criteria, ultimately forming a systematic nursing model comprising the following 7 core modules. The defining feature of this model is that the nursing team assumes a leading or core collaborative role across all modules. The specific modules are detailed below (see Figures 2, 3).

**Figure 2:**
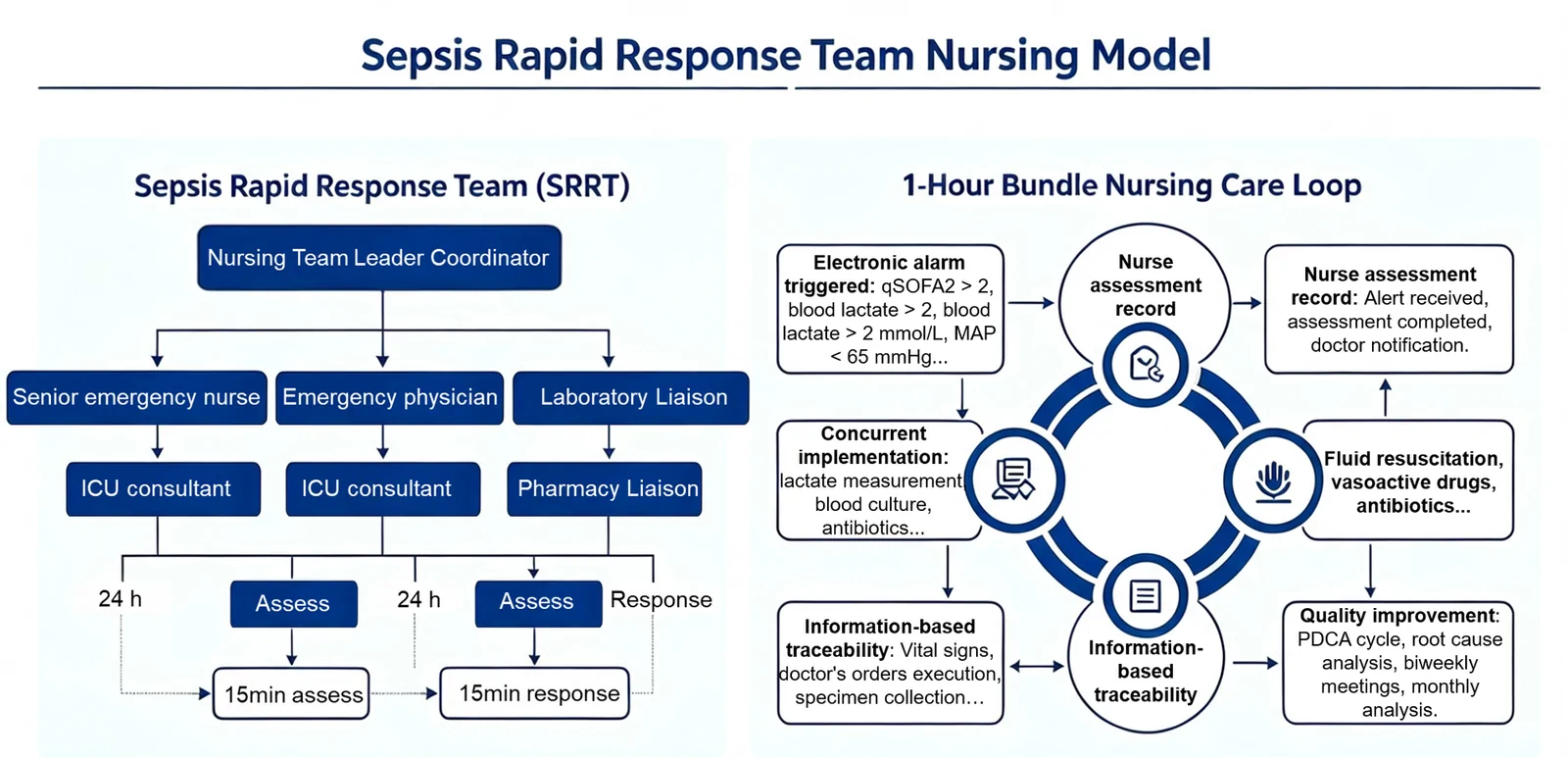
Workflow of the ED nurse-led sepsis rapid response team and 1-hour bundle nursing care loop.

**Figure 3:**
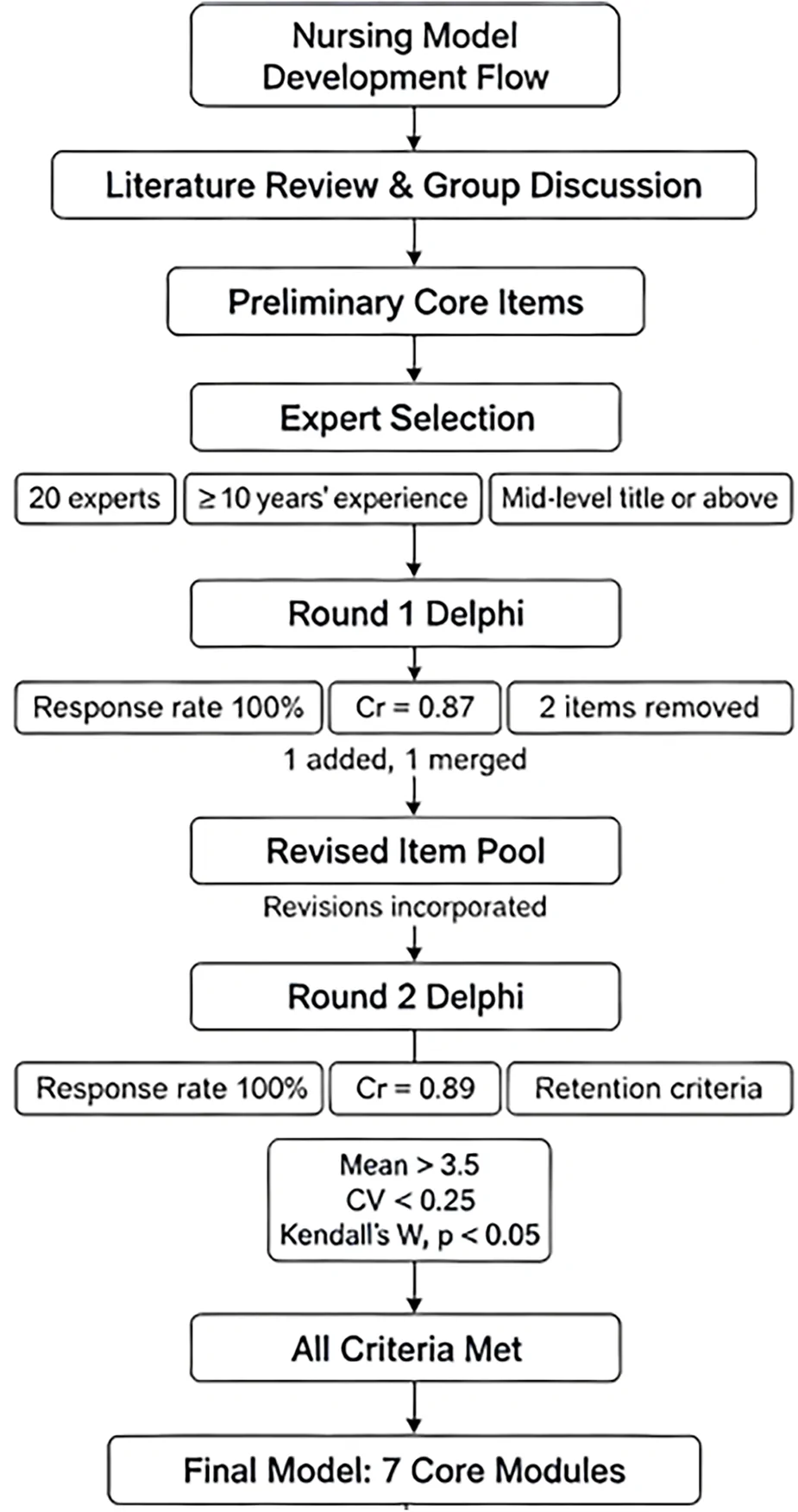
Stepwise formulation of the systematic ED nurse-led septic shock nursing model using the delphi method.

##### 2.3.1.1 Module 1: ED nurse-led sepsis rapid response team (SRRT)

An ED nurse-led SRRT was established, with the nursing team leader serving as coordinator. Team members included senior ED nurses, emergency physicians, ICU consultants, and laboratory/pharmacy liaisons. All nursing staff completed a 2-week systematic training covering Sepsis-3 criteria, SSC 2021 guidelines (3), 1-hour bundle nursing protocols, electronic alarm response, and multidisciplinary communication. They passed both written (≥85%) and practical assessments. The SRRT operated 24/7. Upon receiving an electronic alert, the nurse completed a preliminary assessment within 5 min and notified team members, with core members arriving at the bedside within 15 min.

##### 2.3.1.2 Module 2: nursing trigger and response to the electronic sepsis alert system

A dedicated alarm terminal was installed at the nursing station, interfacing with the hospital HIS/LIS to automatically calculate qSOFA and SOFA scores. Triggers included: 1) qSOFA ≥2; 2) lactate >18 mg/dL with MAP <65 mmHg; or 3) infection-related order sets. Once triggered, the responsible nurse immediately performed a nursing assessment and notified the SRRT physician. Three time points were mandatorily recorded: alarm receipt, assessment completion, and physician notification.

##### 2.3.1.3 Module 3: standardized 1-hour bundle nursing checklist

A checklist was developed for the 1-hour bundle, completed and supervised by the responsible nurse immediately after admission. It included five key elements: 1) lactate measurement–blood sampling within 15 min; 2) blood cultures before antibiotics–bilateral double-bottle collection within 30 min; 3) broad-spectrum antibiotics–preparation and infusion within 1 h; 4) crystalloid resuscitation–rapid dual-channel infusion at 30 mL/kg for hypotension or lactate ≥36 mg/dL; and 5) vasopressor administration–norepinephrine prepared and titrated to maintain MAP ≥65 mmHg via central/peripheral access, with continuous MAP monitoring. Each completed item was checked and timed on the checklist, with dual signatures from the nurse and physician.

##### 2.3.1.4 Module 4: zoned nursing management for patients with abnormal health codes

Upon arrival at triage, nurses checked the patient's health code via the Zhejiang Health Code system. For patients with abnormal codes, nurses immediately activated infection prevention protocols: transferring the patient to an independent isolation resuscitation unit, using dedicated resuscitation carts and protective supplies, assigning a dedicated nurse, and documenting zonal management in nursing records.

##### 2.3.1.5 Module 5: integrated information platform for nursing collaboration

The nursing team led the development of a multi-department information platform covering admissions, imaging, laboratory, pharmacy, and physicians. Using mobile handheld terminals, nurses scanned patient wristbands to record vital signs, confirm medication administration, and verify specimen collection, ensuring full traceability.

##### 2.3.1.6 Module 6: dynamic monitoring-guided precision fluid resuscitation nursing

Four components were implemented: 1) bedside ultrasound to assess inferior vena cava collapsibility (nurse-assisted after training); 2) lactate clearance monitoring every 2 h with timely feedback to physicians; 3) precise fluid input/output recording using electronic urine output monitors and micro-infusion pumps, summarized hourly; and 4) real-time adjustment of vasopressor infusion rates based on MAP values, with documentation of adjustments and effects.

##### 2.3.1.7 Module 7: continuous quality improvement by the nursing quality management group

A hospital-wide sepsis nursing quality improvement working group was established, led by the deputy director of nursing and co-led by the head nurse of emergency medicine, with members including nursing team leaders, quality management representatives, and infection control nurses. The group held bi-weekly routine meetings and monthly quality analysis meetings. Using the PDCA (Plan-Do-Check-Act) cycle, they conducted root-cause analyses for delayed adherence to the bundle, proposed improvement plans, and verified effectiveness in subsequent cycles. The group also oversaw continuing education and re-certification for SRRT nursing members.

#### 2.3.2 Conventional care model

In the Control group, patients with septic shock received treatment and nursing care according to the hospital's routine procedures. Under the conventional model: after admission, patients entered the resuscitation room through the routine triage process; nursing assessment and execution were independently performed by the duty nurse without any standardized sepsis recognition tools; there was no nursing response mechanism for electronic alert systems; no dedicated sepsis rapid response nursing team; no standardized 1-hour bundle nursing checklist; no information system support for the emergency green channel; fluid resuscitation nursing relied mainly on verbal orders from physicians and personal experience; and quality improvement was conducted through routine departmental morning meetings. Both groups received physician-led medical treatment according to institutional protocols and the Surviving Sepsis Campaign guidelines. Sepsis management remained multidisciplinary in both groups. In the control group, patients received routine monitoring, including continuous vital sign monitoring, regular physician assessment, and standard nursing documentation, as per institutional practice during that period. However, the study-specific standardized sepsis follow-up flowchart, electronic alert response, SRRT, 1-hour bundle checklist, and dynamic monitoring protocol were not used in the control group because they had not yet been implemented.

### 2.4 Data collection

#### 2.4.1 Primary outcomes

##### 2.4.1.1 1-hour bundle adherence rate

Defined as the proportion of patients who completed all five core interventions within 1 h after admission: a. measuring lactate levels; b. obtaining blood cultures before antibiotic administration; c. administering broad-spectrum antibiotics; d. administering crystalloid fluid at 30 mL/kg for patients with hypotension or lactate ≥36 mg/dL; and e. administering vasopressors (primarily norepinephrine) to achieve a MAP ≥65 mmHg in patients unresponsive to fluid resuscitation. Nursing checklist records served as the primary source for adherence data.

The adherence rate was calculated as: 1-hour bundle adherence rate = (Number of patients who completed all five elements ÷ Total number of patients in the group) × 100%.

##### 2.4.1.2 28-day and 90-day all-cause mortality

Defined as the proportion of patients who died from any cause from the time of septic shock diagnosis to day 28 and day 90, respectively.

##### 2.4.1.3 24-hour resuscitation success rate

Defined as the proportion of patients who survived (without cardiac arrest or in-hospital death) within 24 h after the diagnosis of septic shock.

#### 2.4.2 Secondary outcomes

1) Dynamic changes in SOFA score. SOFA scores were calculated on day 3 and day 7 after diagnosis and compared with baseline SOFA scores to calculate the magnitude of improvement. SOFA improvement was defined as a decrease of ≥2 points in the day 7 SOFA score from baseline. 2) Duration of vasoactive agent use. The total duration (hours) from the first administration of vasoactive agents to their complete discontinuation with MAP maintained ≥65 mmHg for at least 24 h. 3) Duration of mechanical ventilation. The total duration (days) from the initiation of invasive mechanical ventilation to successful extubation. 4) ICU length of stay. The total duration (days) from ICU admission to ICU discharge or death. 5) Adverse events. The incidence of adverse events related to nursing interventions was recorded in both groups, including: fluid overload (net fluid balance exceeding 10% of body weight within 24 h), antibiotic-related adverse events (acute kidney injury, allergic reactions, etc.), vasoactive agent-related adverse events (arrhythmias, digital ischemia), nausea and vomiting. The incidence of adverse events is expressed as the number of patients (n) and percentage (%) experiencing each specific adverse event, with the denominator being the total number of patients in the group. In Table 5, “Total” refers to the sum of adverse event occurrences across all subgroups (i.e., total number of events), used to reflect the overall burden of adverse events.

#### 2.4.3 Key covariates

Baseline: demographic characteristic and SOFA score. Underlying comorbidities: history of hypertension, diabetes mellitus, chronic kidney disease, chronic heart failure, chronic liver disease, malignancy/hematologic disorders, and immunocompromised status. Site of infection: infectious diseases, respiratory system, cardiovascular system, and others.

### 2.5 Statistical analysis

The statistical work was undertaken with SPSS 26.0 and R 4.1. To obtain propensity scores, we constructed a multivariable logistic regression model, which was executed through the “MatchIt” package available in R, with intervention status as the dependent variable and age, sex, SOFA score, Charlson Comorbidity Index, site of infection, initial lactate level, and antibiotic exposure history as covariates. Propensity score matching was applied to balance observed covariates and reduce confounding. A consultant statistician was involved in the design and interpretation of the PSM analysis. Groups matched 1:1 (nearest neighbor, caliper=0.2SD); balance evaluated by SMD (threshold <0.10). Continuous variables were described as mean ± standard deviation or median (interquartile range). Before matching, intergroup comparisons were performed using independent samples t-test (for normally distributed data) or Mann–Whitney U test (for non-normally distributed data). After matching, paired t-test (for normally distributed data) or Wilcoxon signed-rank test was used. Categorical variables are presented as frequencies (percentages) and compared by chi-square or Fisher's exact test. All hypothesis tests were two-sided, with a significance level of *α*=0.05, and *p* < 0.05 was considered statistically significant.

## 3 Results

### 3.1 Compare the baseline data before and after PSM

Before propensity score matching (PSM), a total of 674 patients were enrolled (331 in the control group and 343 in the observation group). There were significant between-group differences in Antibiotic use (*p* < 0.001), Lactate (*p* = 0.025), APACHE score (*p* = 0.004), and SOFA score (*p* = 0.012), whereas Age, Gender, Disease type, and Charlson Comorbidity Index were comparable (all *p* > 0.05). After PSM, 262 patients were successfully matched in each group (total 524). All variables became well balanced with no statistically significant differences (all *p* > 0.05) see Table 2.

**Table 2:** Compare the baseline data before and after PSM.

Index	Before PSM					After PSM				
	Control group (*n* = 331)	Observation group (*n* = 343)	*Z/χ^2^*	*p*	SMD	Control group (*n* = 262)	Observation group (*n* = 262)	*Z/χ^2^*	*p*	SMD
Age, [year, M (Q_25_, Q_75_)	69.00 (57.00, 79.00)	69.00 (56.00, 76.00)	1.015	0.254	0.073	68.45 (57.00, 78.00)	69.50 (59.00, 76.25)	0.743	0.640	0.011
Gender, *n* (%)			0.359	0.549	0.046			0.032	0.858	0.016
Male	202 (61.03)	217 (63.27)				161 (61.45)	159 (60.69)			
Female	129 (38.97)	126 (36.73)				101 (38.55)	103 (39.31)			
Disease type, *n* (%)			1.994	0.574	0.101			1.725	0.631	0.017
Infectious diseases	165 (49.85)	177 (51.60)				134 (51.15)	126 (48.09)			
Respiratory system	48 (14.50)	57 (16.62)				37 (14.12)	45 (17.18)			
Cardiovascular system	29 (8.76)	32 (9.33)				22 (8.40)	27 (10.31)			
Other	89 (26.89)	77 (22.45)				69 (26.34)	64 (24.43)			
Antibiotic use, *n* (%)	146 (44.11)	221 (64.43)	28.051	<.001	−0.409	135 (51.53)	147 (56.11)	1.106	0.293	−0.092
Lactate, [mg/dL, M (Q25, Q75)	19.82 (12.61, 33.33)	23.42 (13.51, 43.24)	1.483	0.025	−0.145	20.27 (11.71, 33.33)	22.52 (13.51, 39.46)	1.048	0.222	−0.008
APACHE score, [score, M (Q_25_, Q_75_)	19.00 (16.00, 22.00)	20.00 (17.00, 24.00)	1.760	0.004	−0.367	19.00 (16.00, 23.00)	19.00 (17.00, 22.00)	0.481	0.975	−0.020
SOFA score, [score, M (Q_25_, Q_75_)	7.00 (5.00, 9.00)	8.00 (6.00, 11.00)	1.601	0.012	0.289	7.00 (5.00, 9.00)	7.00 (5.00, 10.00)	0.612	0.849	−0.035
Charlson Comorbidity Index, [score, M (Q_25_, Q_75_)	4.00 (3.00, 5.00)	4.00 (3.00, 5.00)	0.491	0.969	0.073	4.00 (2.00, 5.00)	4.00 (3.00, 5.00)	0.306	0.990	0.015

PSM, propensity score matching; SMD, standardized mean difference; M, median; Q25, 25th percentile; Q75, 75th percentile; APACHE, Acute Physiology and Chronic Health Evaluation; SOFA, Sequential Organ Failure Assessment; n, number; %, percentage. Z, Mann–Whitney U test before matching and Wilcoxon signed-rank test after matching; χ^2^, chi-square test.

### 3.2 Comparison of the adherence rates and key outcome indicators

In terms of the adherence of the bundle, the 1-hour bundle adherence rate in the observation group was significantly higher than that in the control group (23.66% vs. 14.12%, *p* = 0.005); however, the absolute adherence rate remained low in both groups. In terms of clinical prognosis, the 24-hour rescue success rate (93.51% vs. 80.92%, *p* < 0.001), 28-day survival rate (81.30% vs. 70.23%, *p* = 0.003), and 90-day survival rate (74.43% vs. 64.89%, *p* = 0.018) of the observation group were all significantly better than those of the control group see Table 3.

**Table 3:** Comparison of the adherence rates and key outcome indicators.

Index	After PSM			
	Control group (*n* = 262)	Observation group (*n* = 262)	*Z/χ^2^*	*p*
1 h bundle adherence rate, *n* (%)			7.784	0.005
Yes	37 (14.12)	62 (23.66)		
No	225 (85.88)	200 (76.34)		
24 h rescue success rate, *n* (%)			18.637	<0.001
Yes	212 (80.92)	245 (93.51)		
No	50 (19.08)	17 (6.49)		
28-day survival rate, *n* (%)			8.740	0.003
Yes	184 (70.23)	213 (81.30)		
No	78 (29.77)	49 (18.70)		
90-day survival rate, *n* (%)			5.643	0.018
Yes	170 (64.89)	195 (74.43)		
No	92 (35.11)	67 (25.57)		

PSM, propensity score matching; 1 h, 1-hour; 24 h, 24-hour; n, number; %, percentage. Z/χ^2^, Wilcoxon signed-rank test or chi-square test as appropriate.

### 3.3 Comparison of nursing effects between the two groups

There were no statistically significant differences in SOFA scores between the two groups on Day 1 and Day 3 (*p* > 0.05); however, the SOFA score on Day 7 was significantly lower in the observation group than in the control group (*p* = 0.002). Regarding hospitalization-related indicators, the observation group had significantly shorter emergency department length of stay, ICU length of stay, total hospital length of stay, and duration of vasoactive agent use compared with the control group (all *p* < 0.05) see Table 4.

**Table 4:** Comparison of nursing effects.

Index	After PSM			
	Control group (*n* = 262)	Observation group (*n* = 262)	*Z*	*p*
Day 1 SOFA score [score, M (Q_25_, Q_75_)	7.00 (5.00, 9.00)	7.00 (5.00, 10.00)	0.612	0.849
Day 3 SOFA score [score, M (Q_25_, Q_75_)	6.00 (4.00, 9.00)	6.00 (4.00, 8.00)	0.786	0.567
Day 7 SOFA score [score, M (Q_25_, Q_75_)	4.00 (2.00, 6.00)	3.00 (2.00, 5.00)	1.835	0.002
Emergency department length of stay [h, M (Q_25_, Q_75_)	10.59 (3.48, 23.88)	7.82 (2.50, 18.03)	1.573	0.014
ICU length of stay [d, M (Q_25_, Q_75_)	2.03 (0.00, 6.73)	1.78 (0.00, 4.06)	1.485	0.024
Total hospital length of stay [d, M (Q_25_, Q_75_)	9.13 (2.79, 15.73)	8.87 (3.73, 13.01)	1.442	0.031
Duration of vasoactive agent use [h, M (Q_25_, Q_75_)	1.08 (0.00, 42.62)	0.00 (0.00, 22.00)	1.573	0.014
Duration of mechanical ventilation [h, M (Q_25_, Q_75_)	0.00 (0.00, 0.00)	0.00 (0.00, 0.00)	0.786	0.567

PSM, propensity score matching; SOFA, Sequential Organ Failure Assessment; M, median; Q25, 25th percentile; Q75, 75th percentile; ICU, intensive care unit; h, hours; d, days. Z, Wilcoxon signed-rank test.

### 3.4 Comparison of adverse reactions

In the control group, there were 12 cases (4.58%) of fluid overload, 15 (5.73%) of allergic reaction, 23 (8.78%) of arrhythmia, 36 (13.74%) of nausea and vomiting, and 0 cases of healthcare-associated infections; the corresponding numbers in the observation group were 5 (1.91%), 8 (3.05%), 7 (2.67%), 14 (5.34%), and 0, respectively. Among these, the incidences of arrhythmia and nausea and vomiting were significantly lower in the observation group than in the control group (*p* < 0.05); whereas the differences in fluid overload, allergic reaction, and healthcare-associated infections were not statistically significant (all *p* > 0.05). Observation group had a lower adverse reaction rate (12.98% vs. 32.82%, *p* < 0.001) see Table 5.

**Table 5:** Comparison of adverse reactions.

Adverse reaction	After PSM			
	Control group (*n* = 262)	Observation group (*n* = 262)	*χ^2^*	*p*
Fluid overload, *n* (%)	12 (4.58)	5 (1.91)	2.979	0.084
Allergic reaction, *n* (%)	15 (5.73)	8 (3.05)	2.228	0.136
Arrhythmia, *n* (%)	23 (8.78)	7 (2.67)	9.052	0.003
Healthcare-associated infections, *n* (%)	0 (0.00)	0 (0.00)	-	-
Nausea and vomiting, *n* (%)	36 (13.74)	14 (5.34)	10.701	0.001
Total, *n* (%)	86 (32.82)	34 (12.98)	29.226	<0.001

PSM, propensity score matching; n, number; %, percentage. χ^2^, chi-square test (or Fisher's exact test when appropriate). The “Total” row represents the sum of all specific adverse event episodes. Patients with multiple events were counted separately in each subcategory.

## 4 Discussion

Septic shock is one of the most common and lethal critical conditions in the emergency department, with a consistently high mortality rate worldwide (14, 15). One-hour bundled treatment has been proven to significantly reduce the risk of organ failure and short-term mortality in patients with sepsis, and is a core quality indicator strongly recommended by current international guidelines (16). However, the implementation of evidence-based guidelines in clinical practice has long been insufficient, and adherence to the 1-hour bundle is generally low in different medical environments (16, 17). This study found that after propensity score matching, the 1-hour bundled adherence rate in the observation group under the systematic nursing model led by emergency department nurses was significantly higher; at the same time, the 24-hour rescue success rate, 28-day and 90-day survival rates were all significantly better than those in the control group. This suggests that a systematic nursing model led by emergency department nurses, integrated with standardized checklist verification, electronic alarm response, and multidisciplinary collaboration, can effectively improve the efficiency of bundled treatment and transform process improvements into definite short-term and medium-term survival benefits.

The 1-hour bundle adherence rate is the core quality indicator of the “Surviving Sepsis Campaign” guideline for sepsis. Its clinical significance lies in the fact that early identification can significantly reduce the risks of organ failure and mortality. In this study, the 1-hour bundle adherence rate of the observation group (23.66%) was higher than that of the control group (14.12%, *p* = 0.005), but the absolute rate remained below 25%, which is lower than the 57.7% reported after a PDSA-based quality improvement initiative in a tertiary ED (18). However, this absolute adherence rate is comparable to those reported in China; among Chinese emergency and critical care populations, 1-hour bundle adherence rates range from 9.95% to 18% (19, 20). In China, the nurse-to-patient ratio in emergency departments is generally low, and each bundle element requires patient consent before execution, making it particularly difficult to complete all five measures within 1 h. Second, limited emergency resuscitation room resources and the early stage of model implementation also contributed to this gap. Nevertheless, our nurse-led systematic nursing model achieved a significant improvement over conventional nursing (from 14.12% to 23.66%), indicating meaningful clinical value in this resource-constrained setting.

The 24-hour rescue success rate reflects the immediate effect of early resuscitation. The observation group (93.51%) was significantly higher than the control group (80.92%, *p* < 0.001), indicating that the acceleration of the adherence of the bundle directly benefited from early fluid resuscitation, antibiotic use, and timely intervention with vasoactive drugs, effectively reversing the hemodynamic disorder in the early stage of shock. The significant improvement in 28-day survival rate (81.30% vs. 70.23%, *p* = 0.003) and 90-day survival rate (74.43% vs. 64.89%, *p* = 0.018) further demonstrated that the survival benefits brought by early standardized treatment could be continuously extended to the long term. Our finding of improved short- and medium-term survival is consistent with a 2022 Japanese study in which in-hospital mortality was 18.0% in bundle-adherent patients versus 30.3% in non-adherent patients (OR = 2.32, 95% CI 1.09–4.95). However, a 7.5-year observational quality improvement study reported a more substantial 90-day mortality reduction (from 64.2% to 45.0%), suggesting that sustained and mature quality improvement programs may achieve greater survival benefits than our early-stage implementation (21). The EGDT trial meta-analysis showed no 90-day mortality benefit (22), but our bundled strategy differs by emphasizing coordinated execution of multiple elements rather than a single process-oriented intervention. A 2026 systematic review and meta-analysis confirmed that 1-hour bundle compliance was significantly associated with reduced short-term mortality (death rate 20.8% vs. 24.7%, 95% CI 0.69–0.84) (23). The 90-day survival difference in our study suggests a potential lasting impact on post-discharge rehabilitation, though this requires confirmation in larger cohorts.

The SOFA score is an internationally recognized tool for assessing the severity of organ dysfunction associated with sepsis (24). This study found that the observation group had lower scores on the seventh day(*p* = 0.002). Our finding that Day 7 SOFA was significantly lower in the observation group is consistent with a 2022 study identifying SOFA trajectory-based subphenotypes, in which the “Rapidly Improving” phenotype had the lowest mortality (5.5%) despite a similar baseline SOFA (25). This supports the concept that dynamic SOFA trajectory, rather than a single time-point value, is a more reliable indicator of organ function recovery and prognosis. Li's meta-analysis included 40 studies involving a total of 59,962 patients, further confirming that the predictive efficacy of SOFA for 28-day or 30-day mortality is significantly better than the initial SOFA (*p* < 0.01) (26). These findings align with our observation that group differences emerged only on Day 7, not Days 1–3, suggesting that clinicians should not prematurely evaluate bundle effects within 48 h.

In addition, the observation group had significantly shorter observation period, ICU stay time, total hospital stay time, and the duration of vasoactive drug use compared to the control group (all *p* < 0.05). These indicators not only reflect the speed of clinical recovery but also directly affect medical costs and bed turnover efficiency (27). The shortened duration of vasoactive drug use indicates that hemodynamic stability was achieved earlier, which may be related to early adequate fluid resuscitation and precise antibiotic treatment reducing microcirculation disorders (28). Delaying the transfer from the emergency department to the ICU was significantly associated with reduced adherence to sepsis bundle, increased mortality, prolonged ventilator use time, and prolonged ICU and hospital stay (29). In this study, the total hospital stay time of the observation group was significantly shorter. From a health economics perspective, this demonstrated the value of the standardized nursing plan, although it might initially increase nursing manpower costs, but later achieved a balance of benefits and costs by reducing ICU occupancy and accelerating recovery. However, there was no statistically significant difference in the mechanical ventilation time between the two groups (*p* > 0.05). This might be because the decision for mechanical ventilation is more influenced by primary lung infection, basic lung function, and ventilator weaning strategy, while the bundle treatment mainly acts on systemic inflammatory response and hemodynamics, having limited impact on the duration of established respiratory support; or it could be that the baseline SOFA scores of the respiratory systems in the two groups were relatively balanced, and the weaning plan was not included in the intervention focus, suggesting that respiratory management could be a supplementary optimization direction for the nursing plan in the future.

In this study, the main differences were manifested in the significant reduction of arrhythmias (2.67% vs. 8.78%) and nausea/vomiting (5.34% vs. 13.74%). The reduction in arrhythmias might be attributed to more meticulous fluid management, electrolyte monitoring, and the standardized use of vasoactive drugs. Previous studies have shown that adrenergic vasopressor drugs are closely related to tachyarrhythmias in septic shock (30); early combined use of vasopressin can reduce the dosage of catecholamines and lower the risk of new arrhythmias (31); a network meta-analysis also indicated that dopamine has the highest risk of arrhythmias, while norepinephrine has a relatively lower risk (32). The decrease in the incidence of nausea/vomiting might be related to the optimization of antibiotic infusion speed and preventive antiemetic measures. The former might be related to the fact that this study only optimized early resuscitation without implementing subsequent restrictive fluid management, while the latter was an individual heterogeneity event of the patient and had a very weak relationship with the speed of nursing execution. Neither group experienced healthcare-associated infections. Overall, the observation group improved the therapeutic effect without increasing safety risks, demonstrating the good safety of this nursing plan.

This study has the following limitations. Firstly, this study is a single-center retrospective matched study with a limited sample size. Although the baseline matching controlled the major confounding factors, there may still be unmeasured residual confounding. The external generalizability of the study results needs to be further verified by multi-center prospective studies. Secondly, the assessment of the 90-day long-term outcome is affected by the completeness of follow-up after discharge. The loss of follow-up for some patients may lead to information bias. Future studies should strengthen the follow-up mechanism and extend the observation window. Finally, this study did not include dynamic monitoring of inflammatory mediators and microcirculation indicators. In the future, the mechanism of organ function recovery can be further explained by combining the trajectory of biomarker changes.

In summary, after balancing the baseline with PSM, the standardized nursing program in the observation group significantly increased the 1-hour adherence rate of the sepsis bundle and translated into a comprehensive improvement in 24-hour rescue success rate, 28-day and 90-day survival rates, while accelerating organ function recovery, shortening hospital stay and the time of using vasoactive drugs, and did not increase the risk of adverse reactions. It is recommended to promote this standardized process in emergency departments and ICUs, and pay attention to the timeliness of implementation to maximize patient benefits.

## Data Availability

The original contributions presented in the study are included in the article/Supplementary Material, further inquiries can be directed to the corresponding author.
